# Tobacco advertising in Points-of-Sale around urban schools in Romania

**DOI:** 10.1186/1617-9625-12-S1-A37

**Published:** 2014-06-06

**Authors:** Vlad Dediu, Lambros Lazuras, Constantine Vardavas

**Affiliations:** 1 Psychology Department, International Faculty of the University of Sheffield, Thessaloniki, 54622, Greece; 2 South East European Research Center, SEERC, Thessaloniki, 54622, Greece; 3 Clinic of Social and Family Medicine, University of Crete, Heraklion, 71003, Greece; 4 Center for Global Tobacco Control, Harvard School of Public Health, Boston, Massachusetts, 02115, USA

## Background

Price promotions in points of sale (POS) are risk factors for tobacco use initiation and shape pro-smoking beliefs among adolescents [1]. The aim of the present study was to assess the extent of tobacco advertisements in POS located near schools in Romania.

## Materials and methods

Tobacco industry advertising was measured in POS (interior and exterior advertising) that were within close proximity (< 300 m) to high schools [2], in the urban area of Bucharest, Romania. A total of 72 POS were identified around 10 schools.

## Results

On average there were 7 POS around each school, with one in twelve POS directly visible from school gates. Advertising was more common internally (77.8% of all POS) than externally, and price promotions were more frequent indoors than outdoors. External tobacco ads were recorded in 19.7% of POS. British American Tobacco and Altria Group, Inc. (parent company of Philip Morris) were responsible for > 60% of external price promotions. Out of the 12 brands recorded, the most widely advertised were Kent, followed by Virginia, Philip Morris and Pall Mall, accounting for 75.7% of the cases. Overall, 36.1% of tobacco advertising was medium-to-high intensity.

## Conclusion

The present study is the first one of its kind conducted in Romania, assessing the geo-position of POS around schools. Internal advertising was more common than external ads or price promotions, and the Altria group was responsible for most of them. The present findings can set the basis for future research into the effects of tobacco advertising around schools on adolescents’ smoking behavior.
